# Cell Adhesion and Matrix Stiffness: Coordinating Cancer Cell Invasion and Metastasis

**DOI:** 10.3389/fonc.2018.00145

**Published:** 2018-05-04

**Authors:** Vasiliki Gkretsi, Triantafyllos Stylianopoulos

**Affiliations:** ^1^Department of Life Sciences, Biomedical Sciences Program, School of Sciences, European University Cyprus, Nicosia, Cyprus; ^2^Cancer Biophysics Laboratory, Department of Mechanical and Manufacturing Engineering, University of Cyprus, Nicosia, Cyprus

**Keywords:** extracellular matrix, cell–extracellular matrix adhesion, actin cytoskeleton, cell invasion, metastasis, stiffness, solid stress, desmoplasia

## Abstract

Metastasis is a multistep process in which tumor extracellular matrix (ECM) and cancer cell cytoskeleton interactions are pivotal. ECM is connected, through integrins, to the cell’s adhesome at cell–ECM adhesion sites and through them to the actin cytoskeleton and various downstream signaling pathways that enable the cell to respond to external stimuli in a coordinated manner. Cues from cell-adhesion proteins are fundamental for defining the invasive potential of cancer cells, and many of these proteins have been proposed as potent targets for inhibiting cancer cell invasion and thus, metastasis. In addition, ECM accumulation is quite frequent within the tumor microenvironment leading in many cases to an intense fibrotic response, known as desmoplasia, and tumor stiffening. Stiffening is not only required for the tumor to be able to displace the host tissue and grow in size but also contributes to cell–ECM interactions and can promote cancer cell invasion to surrounding tissues. Here, we review the role of cell adhesion and matrix stiffness in cancer cell invasion and metastasis.

Cancer cells undergo certain fundamental changes in terms of cell physiology to attain a malignant phenotype. They acquire self-sufficiency in growth signals, insensitivity to growth-inhibitory signals, limitless replicative potential, evasion of apoptosis, sustained angiogenesis, and tissue invasion capacity that enables them to metastasize to distant sites of the body (1, 2). In fact, the latter is the unique “hallmark of cancer” that differentiates benign and malignant tumors and truly defines cancer (3).

Metastasis is a complex process in which cancer cells spread from a primary site to other organs in the body. It consists of several steps and the involvement of the extracellular matrix (ECM), and the cytoskeleton is indisputable. During this process, malignant cells dissociate from the original tumor mass, reorganize their attachment to the ECM though alterations in cell–ECM adhesion dynamics, and start degrading surrounding ECM to eventually invade through adjacent tissues and/or intravasate into blood vessels and travel through the circulation to distant sites of the body (4). The establishment of a metastatic tumor at the new site is not random but rather seems to follow a pattern known as “metastatic tropism.” Cancer cells that have managed to survive in the circulation find a metastatic niche that, based on the “seed and soil” theory, is suitable for their growth (5–7). Hence, some cancer types metastasize according to circulation patterns or based on the anatomical proximity of neighboring organs or the host–organ microenvironment. For instance, prostate cancer shows a preference toward the bone, pancreatic cancer forms metastases to the lung and liver, and breast cancer metastasizes to the bone, liver, lung, and the brain (6–8). Notably, biophysical and biochemical cues from the tumor ECM affect each one of the “hallmarks of cancer” (9) and control cell–cell and cell–ECM adhesions, which in turn determine cancer cell invasion and metastasis (7). Thus, integrins, ECM-related adhesion proteins and cell–cell adhesion proteins play a vital role in regulating the various stages of metastasis and defining the aggressiveness of cancer cells (10).

## Cell–Cell and Cell–ECM Adhesion Proteins in Cancer Cell Metastasis

Cancer cells are able to invade the surrounding ECM in the form of single cells or as collective groups of cells moving together, depending on whether cell–cell adhesion proteins, such as E-cadherin, are completely or partially lost in the original tumor, respectively (11). Although integrin-independent migration has also been described (12), both modes of invasion are considered to be heavily dependent on integrin-mediated adhesion to the ECM, whereas collective invasion also requires dynamic cell–cell adhesions so that loosening of cell junctions becomes sufficient for invasion. Thus, E-cadherin expression or its localization in cell–cell junctions is often lost in advanced cancers and has been linked to higher incidence of metastasis (11).

**Figure 1:**
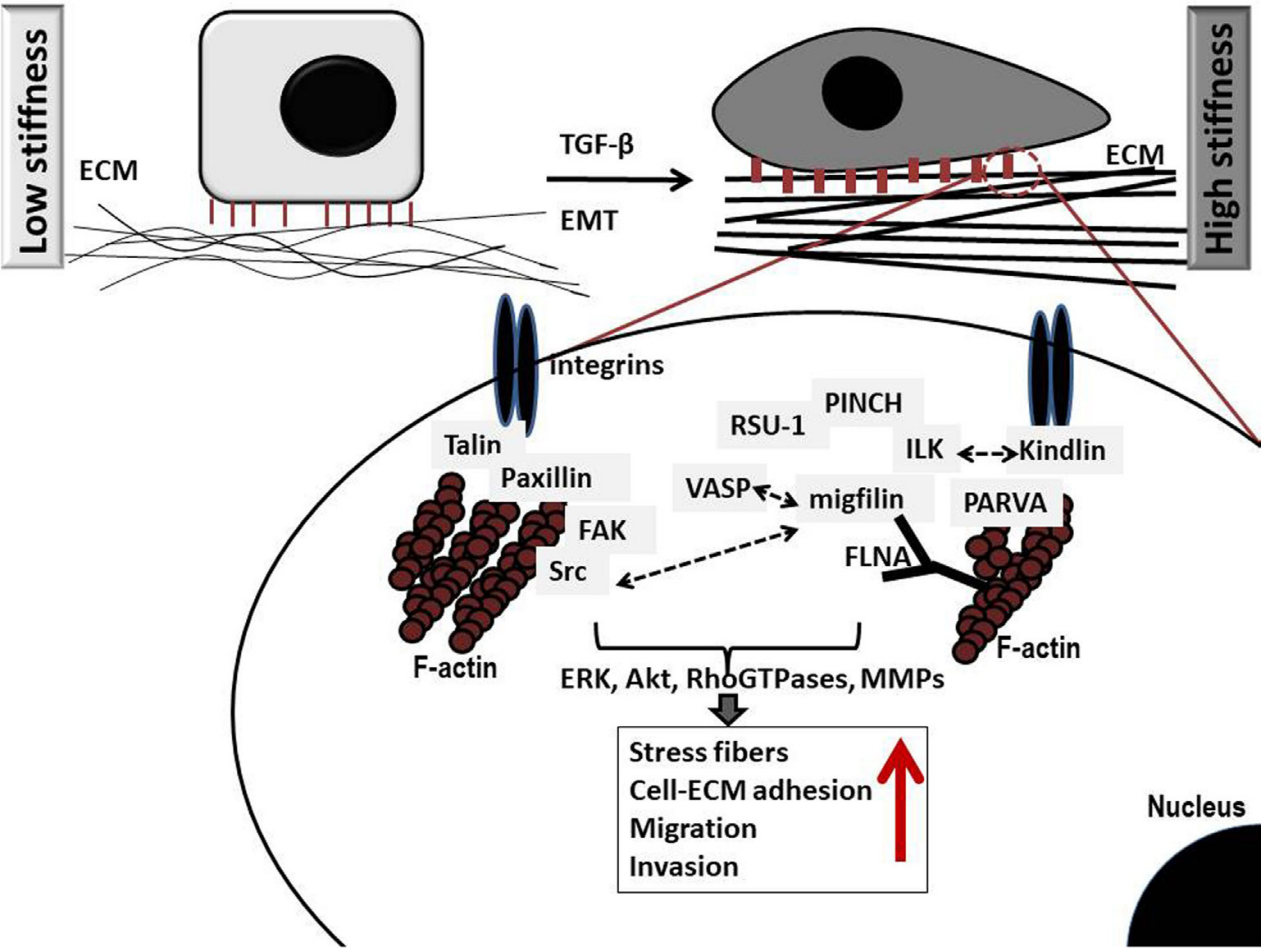
Schematic representation of critical protein–protein interactions at cell–extracellular matrix (ECM) adhesion sites in cancer cells grown in low (left) and high (right) stiffness conditions. Several important protein complexes are formed at the cell–ECM sites that are vital for normal cell function. More specifically, integrin-linked kinase (ILK) binds to the cytoplasmic domain of integrins and also interacts with particularly interesting new cysteine–histidine rich protein (PINCH)-1 and parvin alpha (PARVA) forming a stable ternary complex at cell–ECM adhesions known as PIP (PINCH–ILK–PARVA) or IPP (ILK–PINCH–PARVA) complex (31). PARVA, in turn, binds directly to actin connecting the complex to the cytoskeleton of the cell. ILK has also been shown to interact with Kindlin-2 (also known as mitogen-inducible gene-2 or Mig-2) which again forms a protein complex with Migfilin and filamin A (FLNA) (32), an actin-crosslinking protein. Interestingly, Migfilin has been shown to interact with vasodilator-stimulated phosphoprotein (VASP) (14), regulating cell migration. Equally important is the interaction of integrins with talin (33) and paxillin, which in turn binds to focal adhesion kinase (FAK) (34) while FAK binds to Src (35), which has been also shown to interact with Migfilin regulating cell–ECM mediated survival (36). Note that all cell–ECM adhesion proteins have direct or indirect connection to the actin cytoskeleton, while they activate downstream effectors such as the RhoGTPases, and matrix metalloproteinases (MMPs) eventually leading to regulation of vital cellular functions (proliferation, survival, migration, and invasion). Notably, higher stiffness conditions are associated with marked increase in the amount of stress fibers as well as increased migration and invasion.

However, the actual outcome in terms of invasion is ultimately dependent upon the balance between E-cadherin-mediated adhesions and integrin cell–ECM adhesions (11). Integrins connect the ECM with the interior of the cell transmitting extracellular signals through the assembly of multiple protein complexes that act as adaptor proteins and also bear strong attachments to actin cytoskeleton (10). There are more than 180 cell–ECM proteins forming networks of protein–protein interactions at cell–ECM adhesion sites, which altogether comprise what is known as cell’s *adhesome* (13). Critical determinants in cell–ECM adhesions that also link the ECM directly or indirectly with the actin cytoskeleton include talin, paxillin, kindlins, vinculin, integrin-linked kinase (ILK), parvins [parvin alpha (PARVA), parvin beta, and parvin gamma], particularly interesting new cysteine–histidine rich protein (PINCH)-1, Ras suppressor-1 (RSU-1), vasodilator-stimulated phosphoprotein (VASP) and its interactor Migfilin (14), and α-actinin (15–17). Upon integrin, activation protein tyrosine kinases Src and focal adhesion kinase (FAK) are also activated promoting further cytoskeletal changes as well as activation of downstream signaling pathways vital for cell adhesion, proliferation, survival, migration, and invasion (Figure 1) (18). Small Rho GTPases, Rho, Rac, and Cdc42, as well as Rho-associated protein kinase (ROCK) are such downstream effectors known to coordinate cytoskeletal reorganization and cell migration. Interestingly, most of these components of the cell–ECM adhesions have been found to be significantly deregulated in most cancer types with their expression being associated with higher metastatic potential or lower survival rates (19–26). Moreover, increased levels of RhoA, RhoB, RhoC, Rac1, Cdc42, and ROCK, have been found in late-stage tumors and metastases with prognostic relevance in breast cancer (27, 28). This suggests a strong involvement of cell–ECM adhesion molecules in cancer cell metastasis, although the exact molecular mechanisms involved can be different depending on cell type, tumor location, or grade. In fact, research has shown that cancer cells can have different modes of invasion, and thus a different molecular mechanism activated every time (29, 30). For instance, Rho signaling through ROCK promotes a rounded bleb-associated mode of motility, whereas elongated cell motility is associated with Rac-dependent F-actin-rich protrusions and does not require Rho or ROCK (30).

## Disruption of Cell–Cell Adhesion and Epithelial to Mesenchymal Transition (EMT)

All the above-described changes in cell–ECM and cell–cell adhesion components are important for the detachment of cancer cells from the original tumor mass and their invasion through adjacent tissues and contribute to the “epithelial to mesenchymal transition,” also termed EMT. EMT refers to a transition of polarized epithelial cells toward cells exhibiting mesenchymal properties that enables them to metastasize. Thus, during EMT, epithelial cells reorganize their cytoskeleton, dissociate from one another, and begin expressing mesenchymal genes. These genes may vary significantly in different cells and tissues but there are certain transcription factors, such as TWIST1/2, SNAIL1/2, zinc finger E-box-binding homeobox, and forkhead box protein C2 that are indispensable for EMT in all cases (37–40). In fact, EMT-activating transcription factors have been proposed to have pleiotropic functions acting on all stages of cancer progression from initiation to metastasis (41). Also, several cytokines such as transforming growth factor-β (TGF-β), tumor necrosis factor-α, and interleukin-6, as well as ECM proteins such as collagen I, fibronectin, and hyaluronan are crucially involved in EMT in various tumors (37). Notably, several types of cancer cells have been found to acquire a more mesenchymal-like phenotype which also correlates with their resistance to cytotoxic drugs (38, 42), providing a link between EMT and cancer therapy. Moreover, expression of EMT markers has been also found in circulating tumor cells (CTCs) that are fundamental in the metastatic process. These markers facilitate detection of CTCs while also giving more insights into tumor diagnosis, treatment, and prognosis (43).

All in all, current studies have demonstrated the complexity of the EMT process which raises important and exciting questions for future investigation (41).

## Tumor Microenvironment and Desmoplasia

Apart from cancer cells and the ECM, tumors exhibit an additional aspect of complexity that accounts for the heterogeneity attributed to them and plays an important role in metastasis. They contain a number of allegedly normal cells that comprise the “tumor microenvironment” (1, 44). Hence, structural components of the tumor microenvironment are the tumor blood and lymphatic vessels, and the stromal cell constituents of the tumor that can be subdivided into three categories: (a) angiogenic vascular cells, which include endothelial cells and pericytes, (b) infiltrating immune cells, which include platelets, mast cells, neutrophils, inflammatory monocytes, myeloid-derived suppressor cells (45), macrophages (46), CD8^+^ T-cells, NK T-cells, CD4^+^ T-cells (47), and B cells, and (c) cancer-associated fibroblasts (CAF) cells, which include activated tissue fibroblasts, activated adipocytes, a-smooth muscle actin (α-SMA) positive myofibroblasts, and mesenchymal stem cells (48). As expected, the exact composition of a tumor’s microenvironment varies depending on the tumor type and its location, which justifies the observed heterogeneity among tumors, rendering every tumor unique.

The ECM is a fundamental constituent of the tumor microenvironment that closely interacts with cancer cells for the transmission of signals in and out of the cell through integrins (10), while also providing the necessary growth factors for tumor growth (49). Moreover, upregulation of ECM remodeling molecules, such as TGF-β, are considered to be responsible for the development of *desmoplasia* in tumors (50). Desmoplasia is an intense fibrotic response characterized by the formation of dense ECM consisting of increased levels of total fibrillar collagen, fibronectin, proteoglycans, and tenascin C that accumulates within the tumor. It is associated with increased production and secretion of inflammatory and tumorigenic growth factors, and it is also characterized by an abnormally large population of stromal cells. Moreover, a large percentage of tissue fibroblasts are transformed to CAFs that contain high levels of α-SMA. Therefore, it is proposed that TGF-β activates fibroblasts to become CAFs, which in turn produce more ECM fibers leading to desmoplasia (50). Apart from that, molecules that remodel the ECM, such as matrix metalloproteinases and lysyl oxidase, are also critical for desmoplasia development (51). Collectively, desmoplastic tumors are considered to be more aggressive and are, in fact, associated with worse prognosis in several cancer types (52, 53).

## Role of Tumor Stiffness in Cancer Cell Invasion and Metastasis

Desmoplasia is highly related to tumor *stiffening*, which is perhaps the only mechanical property of tumors that clinicians can really appreciate. Stiffness, which defines how rigid a material is or the extent to which a material resists deformation in response to an applied force (54), depends on the composition and organization of the structural components of a material and describes the extent to which it deforms in response to an applied force or the magnitude of the developed force when the material is subject to a specific strain. Therefore, the stiffer a material is, the more resistant to deformations and more prone to develop higher stresses (i.e., force per unit area) becomes. In tumors, in particular, which are known to grow at the expense of the host tissue, the stress exerted from the tumor on the host should balance the reciprocal stress applied from the host to the tumor. Therefore, the developed stresses within a tumor depend on the relative stiffness between the two tissues and from a biomechanical point of view, stiffening is required for a tumor to be able to displace the host tissue and grow in size (55, 56). Using mathematical modeling, we have previously estimated that tumors should be at least 1.5 times stiffer than their surrounding normal tissue, otherwise confinement by the host prevails to tumor expansion (57).

As mentioned earlier, tumor stiffness is mainly determined by the amount of ECM, particularly collagen and hyaluronan contained in the tumor. Given the fact that the interior of the tumor is subject to compression (58), its stiffness is mainly determined by hyaluronan, which owing to its fixed negative charges creates hydrated, gel-like regions within the tumor capable of resisting compressive stresses (59–62). At the tumor periphery, tumor growth can remodel the collagen fibers and change their orientation toward the tumor circumference. As a result, collagen fibers can be stretched and develop tensile stresses. Therefore, stiffness at the periphery should also depend on the amount of collagen (63, 64).

For the study of ECM stiffness, cancer cells usually grow in three dimensions (3D) within a collagen, hyaluronan, or similar gel that mimics the ECM and parameters that most often vary to modulate stiffness are either the gel’s concentration or the degree of collagen crosslinking for gels that contain collagen. Cancer cell spheroids are also employed for the study of cell invasion through the matrix (65–70). Increasing ECM stiffness has been shown to induce malignant phenotype (71–73) characterized by Rho-dependent cytoskeletal tension that leads to enhanced cell–ECM adhesions, disruption of cell–cell junctions and increased growth (69) (Figure 1) and is actually associated with activated FAK and extracellular signal-regulated kinase signaling (69). Finally, another proof that stiffness is crucially involved in cancer cell metastasis comes from preclinical studies showing that disruption of tumor ECM integrity halts metastasis (74).

Cells can sense ECM stiffening through integrins by cytoskeletal filaments that coordinate cell migration and induce changes within the cell. As that, a stiffer ECM can induce production of fibronectin, a glycoprotein of the ECM that binds from one side to extracellular collagen, fibrin, and heparan sulfate proteoglycans and from the other side to integrins. ECM stiffening can also enhance cell–ECM adhesions that connect the ECM to the cytoskeleton through local adhesion proteins, and increase cytoskeletal tension by Rho/ROCK signaling activation (69, 75). Therefore, integrin clustering can initiate the recruitment of focal adhesion signaling molecules such as FAK, ILK, PARVA, Src, paxillin, as well as Rac, Rho, and Ras that cause cell contractility and can promote tumor progression (Figure 1) (76, 77). In addition, stiffening of the ECM can enhance phosphatidylinositide 3-kinases activity, which regulates tumor invasion (78–80). Furthermore, the cell–ECM adhesion protein RSU-1 was found to be significantly upregulated in increased stiffness conditions in a 3D collagen-based *in vitro* culture system, while tumor spheroids made of cells lacking RSU-1 lost their invasive capacity through the 3D matrix in all stiffness conditions (65). Moreover, lack of the actin polymerization regulator VASP, also inhibited tumor spheroid invasion through matrix of increasing stiffness indicating that both actin cytoskeleton and cell–ECM adhesions play pivotal role in tumor spheroid invasion through 3D matrix (81), an *in vitro* property that mimics tumor invasion in a real tumor setting.

In addition, in pancreatic tumors with mutant SMAD4, matrix stiffening was associated with elevated ROCK activity that in turn stimulated increased production of ECM, assembly of focal adhesions and signal transducer, and activator of transcription-3 (STAT-3) signaling driving tumor progression (82). Matrix stiffening can also induce EMT, leading to the acquisition of a more aggressive phenotype that promotes cancer cell invasion owing to a loss of intercellular adhesions (83), and it is hypothesized to contribute to the transformation of cancer cells to stem cell-like cancer cells that can survive under the harsh hypoxic conditions of the tumor microenvironment, are more resistant to cytotoxic drugs, and can migrate and invade through surrounding tissues (84).

## Effects of Solid Stress on Cancer Cell Behavior

It should be noted, however, that even though ECM stiffness can be related to the magnitude of solid stress, the two quantities are distinct and thus, one should not be used to replace the other (85). Solid stress is defined as the force per unit area of the structural components of a tissue, which can cause either compaction (compression) or expansion (tension) of the material, whereas stiffness refers to the extent to which the tissue can resist deformations or external forces (54).

Different experimental procedures have been also developed to study the effects of solid stress and ECM stiffness on cancer cell behavior. For the study of solid stress, transmembrane pressure devices, cancer cell spheroids, or modifications of these are most often used. In transmembrane pressure devices, cells grow as single cells embedded in a matrix or as a monolayer on a transwell insert membrane, and a piston with adjustable weight is placed on the top to apply a predefined stress (86–89). This method has been used to study stress induced changes in gene expression, invasion, and migration. In the tumor spheroid model, cancer cell aggregates form spheroids that are embedded in a matrix that mimics tumor ECM, such as agarose, collagen, or matrigel. The matrix exerts an external stress to the cells and pertinent studies focus on the effect of solid stress on cancer cell proliferation and apoptosis (87, 90–93). This method is limited, however, in that the applied by the matrix stress cannot be directly quantified. When applied to compress cancer cells, solid stress can inhibit proliferation, induce apoptosis, and increase cancer cell invasive and metastatic potential (87, 88, 90). Compressive solid stress can also activate fibroblasts to become CAFs (similarly to TGF-β), which in turn can facilitate not only development of desmoplasia but also cancer cell invasion to the surrounding, normal tissues (89, 94).

## Concluding Remarks and Perspectives

Cell–ECM adhesion proteins, actin cytoskeleton, and ECM stiffness evidently play a major role in driving cancer cell invasion and metastasis being involved in virtually all steps of the metastatic process from cell dissociation from the original tumor, to invasion through surrounding ECM until the final step of cancer cell homing in the new metastasis site. For this to happen, ECM is connected through integrins to the cell’s adhesome at cell–ECM adhesion sites where multiple protein–protein interactions take place connecting the ECM to the actin cytoskeleton so that response to external stimuli is well coordinated. In fact, signals from these cell-adhesion proteins appear to be crucial for defining the invasive potential of cancer cells, while evidence shows that they may also prove potent targets for inhibiting cancer cell invasion and thus, metastasis (65, 81). Moreover, as ECM stiffness is also a driving force in metastasis (72, 73), it also needs to be taken into account when studying cancer cell metastasis both *in vitro* and *in vivo* in an attempt to better recapitulate tumor microenvironment in a physiologically relevant manner. Thus, the development of appropriate and physiologically relevant *in vitro* systems is needed to define the molecular determinants in the process and open new avenues in the discovery of novel therapeutic candidates to block metastasis.

From another point of view, solid stress is a distinct parameter that affects cancer cell behavior and should be also considered in *in vitro* tumor models. Furthermore, solid stress is exerted not only on cancer cells but also on the endothelial cells that form the tumor micro-vessels. As a result, blood vessels can be compressed or totally collapsed, creating large avascular regions within the tumor thus causing hypo-perfusion and hypoxia (58, 95) which ultimately inhibit systemic administration of drugs to the tumors (55, 60) and can promote tumor progression in multiple ways (96). Notably, recent *in vivo* evidence has shown that modulating the tumor microenvironment through administration of drugs that alleviate intratumoral solid stress (such as anti-fibrotic agents) reduces mechanical stresses, decompresses tumor vessels, and improves tumor drug delivery (60–62, 97), once again suggesting that modulation of ECM is of fundamental significance for tumor biology and cancer therapeutics.

## Author Contributions

Both authors contributed equally to writing, editing, and approving the final version of this article.

## Conflict of Interest Statement

The authors declare that the research was conducted in the absence of any commercial or financial relationships that could be construed as a potential conflict of interest.

## Abbreviations

3D, three dimensional; CAF, cancer-associated fibroblasts; CTC, circulating tumor cell; ECM, extracellular matrix; EMT, epithelial to mesenchymal transition; ERK, extracellular signal-regulated kinase; FAK, focal adhesion kinase; FLNA, filamin A; FOXC2, forkhead box protein C2; ILK, integrin-linked kinase; LOX, lysyl oxidase; MDSC, myeloid-derived suppressor cell; MMP, matrix metalloproteinase; MSC, mesenchymal stem cell; PARVA, parvin alpha; PARVB, parvin beta; PARVG, parvin gamma; PI3K, phosphatidylinositide 3-kinase; PINCH, particularly interesting new cysteine-histidine rich protein; RSU-1, Ras suppressor-1; ROCK, Rho-associated protein kinase; SMA, smooth muscle actin; STAT, signal transduced and activator of transcription; TGF-β, transforming growth factor-β; TNF-α, tumor necrosis factor-α; VASP, vasodilator-stimulated phosphoprotein; ZEB1/2, zinc finger E-box-binding homeobox.
